# Field Investigation and Molecular Detection of Pigeon Paramyxovirus‐1 (PPMV‐1) in Domestic Pigeons (*Columba livia*) From Selected Egyptian Governorates

**DOI:** 10.1155/vmi/3626360

**Published:** 2026-07-25

**Authors:** Muhammadtaher Abdulrazaq Abdulrasol, Wafaa A. Abd El-Ghany, Harith Abdulla Najem

**Affiliations:** ^1^ Department of Pathology and Poultry Diseases, College of Veterinary Medicine, University of Basrah, Basrah, Iraq, uobasrah.edu.iq; ^2^ Poultry Diseases Department, Faculty of Veterinary Medicine, Cairo University, Giza, Egypt, cu.edu.eg

**Keywords:** Egypt, neurotropic strain, pigeon paramyxovirus-1 (PPMV-1), RT-PCR, virus isolation

## Abstract

Pigeon paramyxovirus‐1 (PPMV‐1) is a pigeon‐host variant of avian paramyxovirus type 1 (APMV‐1) associated with neurological, enteric, and respiratory disease in pigeons. This study aimed to investigate the circulation of PPMV‐1 among domestic pigeons (*Columba livia*) in some Egyptian governorates through the clinico‐pathological picture, isolation, and molecular identification. A total of 39 domestic pigeons (*Columba livia*), aged 3–14 months, were collected between April and September 2025 from live bird markets and private lofts in seven Egyptian governorates. Suspected pigeons were examined for the specific clinical signs and gross postmortem lesions, and then pooled tissue samples were processed for PPMV‐1 isolation in embryonated chicken eggs (ECEs) and detection by reverse transcription polymerase chain reaction (RT‐PCR). Neurological signs, including torticollis, head tremors, and circling, were recorded in more than 60% of cases, alongside greenish diarrhea and conjunctivitis. Gross lesions included brain hemorrhages, enlarged kidneys with petechiae, hepatic congestion, proventricular hemorrhages, and pulmonary congestion. Virus inoculation in ECEs caused embryo mortality within 48–72 h with severe hemorrhages and growth retardation. RT‐PCR confirmed the presence of PPMV‐1 RNA in 10 out of 39 samples (25.6%) from Cairo, Giza, Qalyubia, Fayoum, Minya, and Alexandria governorates. This study provides field and laboratory evidence of PPMV‐1 circulation in Egyptian pigeons and underscores the importance of surveillance and biosecurity to limit virus spread. Further molecular characterization, including sequencing and phylogenetic analysis, is recommended in future studies to better define the evolutionary relationships of the circulating strains.

## 1. Introduction

Order Mononegavirales, family Paramyxoviridae, comprises a wide range of enveloped viruses with nonsegmented, single‐stranded, negative‐sense RNA genomes. Paramyxoviridae members are of particular medical and veterinary importance due to infection of various vertebrates and frequent display of marked host specificity [1, 2]. Avian paramyxovirus type 1 (APMV‐1), classified within the species *Avian orthoavulavirus 1*, belongs to the family *Paramyxoviridae*, subfamily Avulavirinae, and genus *Orthoavulavirus*. Virulent APMV‐1 strains are the causative agents of Newcastle disease (ND) in poultry.

ND is recognized by the World Organisation for Animal Health (WOAH) as a disease of major economic and trade significance, and APMV‐1 has been reported in more than 240 avian species, including domestic and wild pigeons [3, 4]. The genome of APMV‐1 ranges from 150 to 250 nm in size and approximately 15 kb encoding fusion (F), hemagglutinin‐neuraminidase (HN), matrix (M) protein, nucleoprotein (N), phosphoprotein (P), and large polymerase (L) proteins [5]. Additionally, APMV‐1 is divided into two genetically distinct classes, I and II, and class II currently comprises 20 genotypes according to the updated unified phylogenetic classification system [5]. The fusion protein is a major determinant of virulence and pathogenicity [3, 5].

Pigeon paramyxovirus type 1 (PPMV‐1), a host‐adapted variant of APMV‐1 Genotype VI, is associated with disease and ND‐like clinical signs in feral and domestic pigeons (Columbidae). Cross‐infection to other avian species has also been reported and may pose a continued threat to poultry production [6, 7]. Strains of PPMV‐1 exhibit a wide range of different clinical pictures, including nervous (tremors of the neck and wings, bilateral or unilateral locomotor disorders, torticollis, paralysis, and disturbed equilibrium), digestive (polydipsia, polyuria, anorexia, and greenish diarrhea), and respiratory distress (gasping, coughing, sneezing, and tracheal rales) with up to 90% mortalities in the infected population [6, 8, 9]. This virus could replicate in different tissues, mainly the brain, lung, kidney, trachea, spleen, liver, bursa of Fabricius, and pancreas [10].

PPMV‐1/APMV‐1 infection in pigeons has been documented in Egypt for decades, and several outbreaks have been reported in different governorates [11–13]. The virus is now considered enzootic in Egypt and continues to cause recurrent outbreaks and economic losses in pigeons and, potentially, in nearby poultry populations [14]. In the broader Middle East, PPMV‐1 was first isolated in Iraq in 1978, marking the onset of its regional spread and subsequent dissemination to other continents [15–17]. By the early 1980s, outbreaks had also been reported in Europe and North America, occasionally spreading to domestic poultry flocks [18, 19].

Proper and effective prevention and control measures, in addition to diagnostic techniques, are crucial for protecting pigeons and other bird species from PPMV‐1 [20]. While vaccination of racing pigeons against ND is compulsory in many countries, pigeon‐rearing strategies in Egypt vary and do not necessitate routine immunization; therefore, they are at greater risk of infection [6, 14]. Therefore, improved surveillance, biosecurity, and evaluation of appropriate vaccination programs may help reduce the disease burden in Egypt.

Field diagnosis of PPMV‐1 infection in pigeons can be challenging because its neurological and enteric manifestations may overlap with other conditions such as avian herpesvirus infection, thiamine (vitamin B1) deficiency, toxicosis, or bacterial diseases like salmonellosis [11, 16]. Therefore, laboratory confirmation through virus isolation and molecular detection remains essential to avoid misdiagnosis [4, 21]. In this regard, exclusion of *Salmonella* infection, which can cause nervous manifestations and diarrhea in pigeons, represents an important step in the differential diagnosis of suspected PPMV‐1 as the primary etiological agent.

The current study was therefore designed to investigate suspected field cases of PPMV‐1 infection in domestic pigeons (*Columba livia*) from selected Egyptian governorates through clinical and pathological examination, virus isolation in embryonated chicken eggs (ECEs), and preliminary molecular confirmation using RT‐PCR.

## 2. Materials and Methods

### 2.1. Ethical Approval

All management procedures and experimental protocols were conducted and reviewed in accordance with the regulations and ethical guidelines of the Animal Care and Use Committee, Faculty of Veterinary Medicine, Cairo University (Vet CU110520251158).

### 2.2. Pathological Examination and Sample Processing

A total of 39 domestic pigeons (*Columba livia*), aged 3–14 months, were collected between April and September 2025. Pigeons exhibiting neurological and gastrointestinal signs were obtained from live bird markets and private lofts across seven Egyptian governorates, including Cairo, Giza, Qalyubia, Fayoum, Beni Suef, Minya, and Alexandria. Suspected pigeons were either found dead or humanely euthanized before sampling. Each bird underwent a thorough clinical examination and necropsy.

A total of 195 pooled tissue samples, 5 organs (brain, lung, kidney, liver, and trachea), were aseptically collected from each pigeon. Tissue samples were homogenized in phosphate‐buffered saline (PBS; pH 7.0–7.4) supplemented with amikacin (100 μg/mL) and fluconazole (20 μg/mL), using sterile sand (0.1‐0.2 g per gram of tissue). The homogenates were centrifuged at 3000 rpm for 10 min at 4°C, and supernatants were stored at −80°C for further analyses.

Bacteriological examination was performed in addition to viral investigation for the possible presence of *Salmonella* species. Briefly, tissues were inoculated onto xylose lysine deoxycholate (XLD) agar and incubated at 37°C for 24–48 h. The absence of characteristic *Salmonella* colonies was used to exclude bacterial infection as a differential diagnosis in pigeons showing neurological and enteric signs.

### 2.3. Virus Isolation in ECEs

Specific pathogen‐free (SPF) ECEs (9–11 days old) were obtained from Nile SPF Company, Fayoum, Egypt. After centrifugation of the prepared tissue supernatants, 0.2 mL of the clarified fluid was filtered through a 0.2‐μm Millipore filter and inoculated into the allantoic cavity of five eggs per sample (*n* = 5 eggs/sample), following the standard allantoic route [4]. The inoculated eggs were incubated at 37°C and monitored daily for 4 days. Eggs exhibiting embryonic death within the first 24 h postinoculation were discarded. The remaining eggs, after 4 days of incubation, were refrigerated at 4°C for 4 h before harvesting the allantoic fluid. All harvested allantoic fluids were stored at −80°C until further use for molecular detection.

### 2.4. RT‐PCR and Preliminary Molecular Detection

Viral RNA was extracted from the infected allantoic fluid of inoculated eggs using the QIAamp Viral RNA Mini Kit (QIAGEN, Cat. No. 52904) according to the manufacturer’s instructions (Qiagen Handbook). Briefly, 140 μL of the sample was mixed with 560 μL of Buffer AVL (viral lysis buffer with carrier RNA) and thoroughly vortexed. After incubation at room temperature for 10 min, 560 μL of ethanol was added to the mixture. The solution was then applied to QIAamp spin columns for RNA binding, followed by sequential washing with Buffer AW1 (Wash Buffer 1) and Buffer AW2 (Wash Buffer 2). Finally, the RNA was eluted in 60 μL of Buffer AVE (elution buffer, RNase‐free) and stored at −80°C until further analysis.

One‐step RT‐PCR was performed using the QuantiTect Probe RT‐PCR Kit (QIAGEN, Cat. No. 204443) according to the manufacturer’s instructions (Qiagen Handbook, January 2008). The amplification reaction was carried out in a final volume of 25 μL, composed of 12.5 μL of 2× QuantiTect Probe RT‐PCR Master Mix, 0.5 μL each of the forward and reverse primers, in (Table 1), (20 pmol; Metabion, Germany), 0.125 μL of QuantiTect RT Mix, 6.375 μL of RNase‐free water, and 5 μL of extracted RNA template.

**TABLE 1:** Primers used for RT‐PCR amplification of the PPMV‐1 F gene.

Name	Gen/primer	Primer sequence (5′–3′)	Size (bp)	Reference
PPMV‐1	F gen/(F)	CAGCTGCGGCCCTAATACA	486	Chang et al. [22]
	F gen/(R)	TGGATGCCCAAGAGTTGAG	486	Chang et al. [22]

*Note:* PPMV‐1: pigeon paramyxovirus‐1; (F): forward primer; (R): reverse primer; bp: base pairs (product size in nucleotides).

Abbreviation: F gen, fusion gene.

Thermal cycling was performed in a Biometra T3 thermal cycler under the following conditions [22]: reverse transcription at 50°C for 30 min, initial denaturation at 95°C for 15 min, followed by 35 cycles of denaturation at 94°C for 30 s, annealing at 55°C for 40 s, and extension at 72°C for 45 s, with a final extension at 72°C for 10 min. The amplified product corresponded to a 486 base pair (bp) fragment of the fusion (F) gene of PPMV‐1.

### 2.5. Gel Electrophoresis

Amplified products were resolved on a 1.5% agarose gel prepared in 1 × TBE buffer [23]. Ethidium bromide (0.5 μg/mL) was incorporated into the gel for nucleic acid staining. A 100‐bp DNA ladder (GeneRuler, Cat. No. SM0243, Fermentas) was used as a molecular weight marker. Twenty microliters of each RT‐PCR product were loaded alongside positive and negative controls. Electrophoresis was performed at 5 V/cm for ∼30 min, and the gels were visualized under UV light using a gel documentation system (Alpha Innotech). Digital images were captured and analyzed using imaging software.

### 2.6. Statistical Analysis

The current study was mainly descriptive. Microsoft Excel 365 was used to summarize the data as counts and percentages. No inferential statistical tests were performed.

## 3. Results

### 3.1. Clinical and Pathological Examination

During the surveillance period (April–September 2025), a total of 39 domestic pigeons, aged 3–14 months, were examined for clinical and pathological manifestations consistent with PPMV‐1 infection. A detailed summary of case history, clinical signs, and gross pathological lesions observed in the examined pigeons is presented in Table 2.

**TABLE 2:** Summary of clinical history, signs, and gross lesions in pigeons suspected of PPMV‐1 infection in Egyptian governorates.

Case no.	Age (months)	Location (governorate)	Clinical signs	Gross lesions
1–7	3–11	Cairo (El‐Marg, Sayeda Aisha, Basateen, Zarayeb, Misr El‐Tairan)	Tremors, torticollis, stargazing, conjunctivitis, diarrhea, circling	Brain hemorrhages, renal enlargement with petechiae, hepatomegaly, lung congestion, proventricular hemorrhage
8–12	6–10	Giza (Osim, Imbaba)	Tremors, circling, weakness, diarrhea	Brain hemorrhages, kidney enlargement, lung congestion, proventricular hemorrhage
13–15	6–9	Fayoum	Stargazing, greenish diarrhea	Brain hemorrhage, renal enlargement, lung congestion
16–19	6–12	Qalyubia	Head tremors, circling, diarrhea	Kidney petechiae, hepatomegaly, lung congestion
20–24	6–10	Minya	Neurological signs (torticollis, tremors), diarrhea	Brain hemorrhage, liver congestion, renal lesions
25–29	8–14	Alexandria	Torticollis, conjunctivitis, diarrhea	Brain hemorrhage, kidney enlargement, pulmonary congestion
30–31	7–12	Beni Suef	Neurological signs, diarrhea	Brain hemorrhage, renal lesions

**FIGURE 1:**
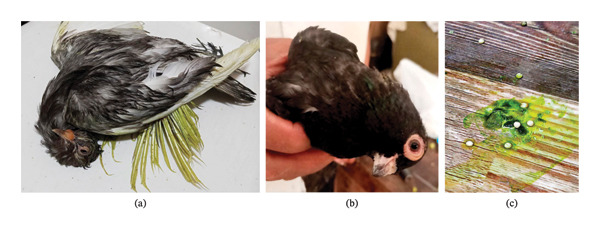
Clinical signs in a 9‐month‐old domestic pigeon naturally infected with pigeon paramyxovirus‐1. (a, b) Torticollis and loss of equilibrium; (c) greenish diarrhea.

**FIGURE 2:**
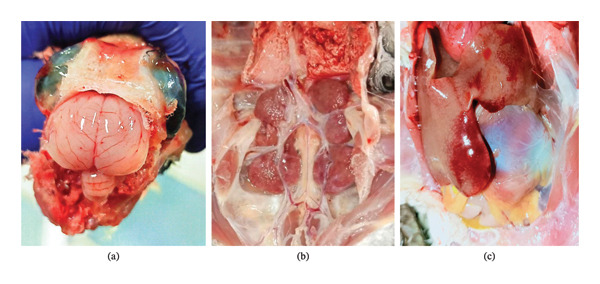
Gross lesions of a 9‐month‐old domestic pigeon naturally infected with pigeon paramyxovirus‐1. (a) Multiple hemorrhages on the brain. (b) Kidney enlargement with petechiae. (c) Enlarged livers with surface hemorrhages.

The most frequently recorded clinical signs included torticollis, head tremors, circling movements, conjunctivitis, and greenish diarrhea (Figure 1), while mild respiratory manifestations such as rales and dyspnea were less common. Postmortem examination consistently revealed characteristic lesions, namely cerebral petechial hemorrhages, renal enlargement with multifocal petechiae, hepatic congestion with surface hemorrhages (Figure 2), proventricular hemorrhages, and pale or congested lungs.

**FIGURE 3:**
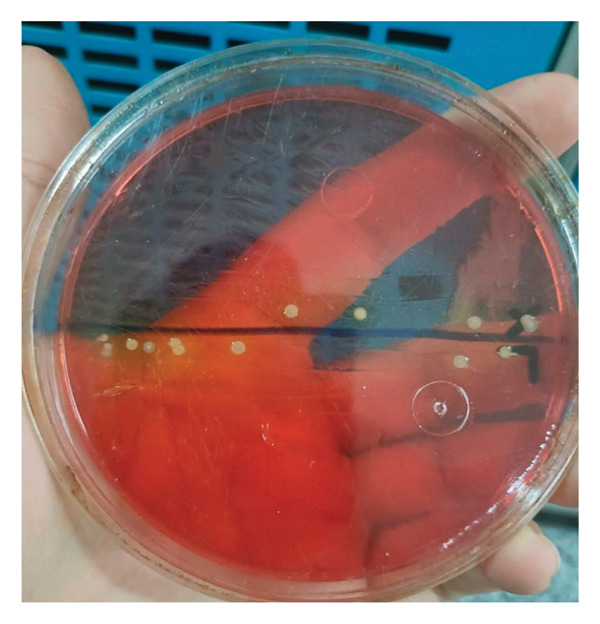
Bacteriological culture results on XLD agar showed no characteristic *Salmonella* growth from pooled organ samples of suspected pigeons.

Bacterial examination of pooled organ samples on XLD agar revealed that all suspected pigeons were negative for *Salmonella* spp., thereby helping to exclude salmonellosis as a differential diagnosis for the observed neurological and enteric manifestations. Representative culture plates showed no characteristic *Salmonella* growth on XLD agar (Figure 3). This finding supports the interpretation that *Salmonella* spp. were not involved in the examined cases.

### 3.2. Virus Isolation in ECEs

**FIGURE 4:**
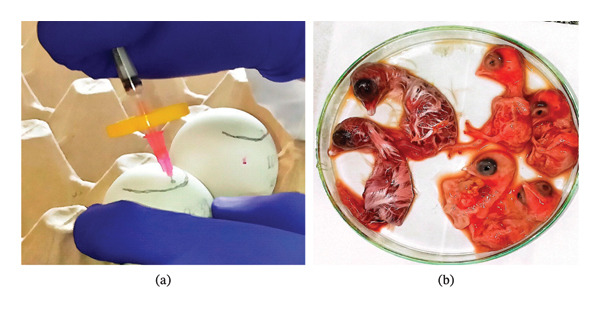
Pigeon paramyxovirus‐1 isolation in embryonated chicken eggs. (a) Inoculation of prepared infected tissue supernatants into 9–11‐day‐old SPF embryonated chicken eggs. (b) Infected embryos (right side) showed marked growth retardation, and diffuse subcutaneous hemorrhages in contrast with normal embryos from uninoculated control eggs (left side).

Tissue homogenates from all 39 pigeons were inoculated into the allantoic cavity of 9–11‐day‐old SPF ECEs. Embryonic changes were recorded after 4 days postinoculation, as the infected embryos showed marked growth, retardation or stunting, incomplete development, and diffused subcutaneous hemorrhages, particularly in the head, neck, and abdominal regions. In contrast, embryos from uninoculated control eggs developed normally without hemorrhages or stunting (Figure 4). After 4 days of incubation, allantoic fluid was harvested from viable embryos and stored at −80°C until further analysis.

### 3.3. RT‐PCR and Molecular Detection

To confirm the presence of PPMV‐1 at the molecular level, RT‐PCR targeting the conserved region of the F gene was performed on RNA extracted from harvested allantoic fluids obtained after inoculation of pooled tissue homogenates into ECEs. RT‐PCR results confirmed the presence of PPMV‐1 RNA in 10 tested samples out of 39 pigeons (25.6%), with positive cases distributed across six governorates: Cairo, Giza, Fayoum, Qalyubia, Minya, and Alexandria (Table 3).

**TABLE 3:** Summary of RT‐PCR results for PPMV‐1–positive pigeon samples.

No.	Sample ID	Governorate	RT‐PCR result
1	5	Cairo	+
2	9	Giza	+
3	19	Cairo	+
4	21	Cairo	+
5	25	Fayoum	+
6	28	Qalyubia	+
7	34	Minya	+
8	37	Alexandria	+
9	38	Alexandria	+
10	39	Alexandria	+

*Note:* +: Positive.

**FIGURE 5:**
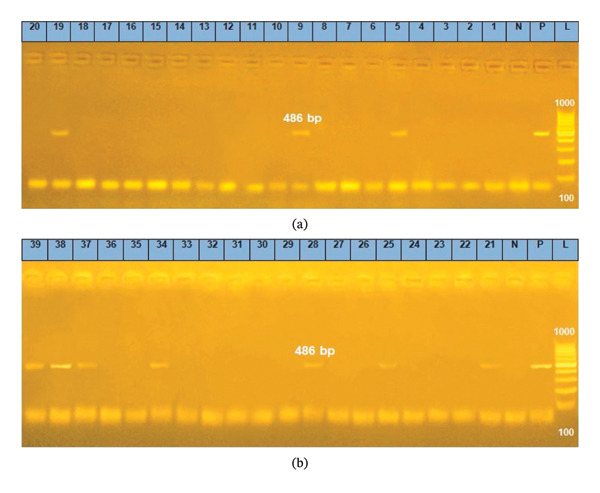
RT‐PCR amplification of the PPMV‐1 fusion (F) gene (486 bp). (a) Samples 1–20: positive bands in Lanes 5, 9, and 19. (b) Samples 21–39: positive bands in Lanes 21, 25, 28, 34, 37, 38, and 39. Abbreviations: P: positive control; N: negative control; L: 100 bp DNA ladder.

Gel electrophoresis of the amplified products on 1.5% agarose revealed distinct bands of the expected size (486 bp) in all positive samples, while no amplification was detected in negative controls. Figure 5 shows the RT‐PCR profiles for Samples 1–20 and 21–39, respectively. Positive bands could be observed in Lanes 5, 9, and 19 (Figure 5a) as well as in Lanes 21, 25, 28, 34, 37, 38, and 39 (Figure 5b). Both the positive (P) and negative (N) controls performed as expected, confirming the assay’s reliability.

## 4. Discussion

The present study provides both field and laboratory evidence of PPMV‐1 circulation among domestic pigeons (*Columba livia*) in some Egyptian governorates. A diagnostic approach combining specific clinical signs and characteristic gross lesions, virus isolation in ECEs with typical embryonic changes, and RT‐PCR targeting the F gene was successfully applied for the preliminary detection of PPMV‐1 [10]. This multistep diagnostic strategy agrees with previous recommendations by Cattoli [8] and WOAH [4], who emphasized the importance of combining clinical, pathological, and molecular tools for accurate identification of PPMV‐1.

In addition, the exclusion of *Salmonella* spp. strengthened the diagnostic approach, ruling out an important bacterial differential diagnosis. This aspect is of diagnostic significance, as salmonellosis in pigeons is well documented to produce nervous manifestations and diarrhea that may confound PPMV‐1 diagnosis. The bacteriological findings in the present study support the interpretation that *Salmonella* spp. were not involved in the examined cases.

Clinically, the affected pigeons exhibited characteristic neurological signs, including torticollis, head tremors, circling, and stargazing, as well as greenish diarrhea and conjunctivitis. These findings are consistent with the previous reports of velogenic neurotropic PPMV‐1 strains [11, 24]. Further, the gross postmortem pathological findings, including brain hemorrhages, renal enlargement with petechiae, hepatic congestion, and proventricular and pulmonary lesions, are consistent with previously reported pathogenic PPMV‐1 infections in pigeons, as shown by Al‐Mubarak [24]. These clinical and pathological findings are strongly indicative of infection with a neurotropic velogenic pathotype of PPMV‐1 [10]. Such overlapping neurological and visceral lesions reflect the multiorgan tropism of PPMV‐1, and their occurrence in > 60% of the studied pigeons confirms the circulation of virulent strains comparable to those described in Saudi Arabia and Iraq [6, 17].

The inoculation of suspected samples in ECEs resulted in embryos’ mortality accompanied by growth retardation and diffuse hemorrhages, which are characteristic indicators of ND virus (NDV)/PPMV‐1 replication [6]. These embryonic changes further confirm the high virulence of the field isolates, in line with earlier observations by Kaleta et al. [15] and Mansour et al. [14], who demonstrated similar embryo pathology with velogenic PPMV‐1 strains.

Subsequent RT‐PCR amplification of the F gene confirmed the presence of PPMV‐1 RNA in 10 out of 39 tested samples, yielding the expected 486 bp amplicon. This finding emphasizes the high specificity and reliability of molecular assays for the preliminary confirmation of PPMV‐1 infection in pigeons and is consistent with previous reports [7, 22]. The detection rate (25.6%) in the present study is comparable to previous reports in Egypt and other regions, which ranged from 20% to 35% positivity [13, 14]. Differences among governorates may be attributed to variable pigeon densities, husbandry practices, and biosecurity levels, with higher positivity in Cairo and Alexandria reflecting the role of live bird markets as focal points for virus transmission.

Overall, the present findings support the continued circulation of PPMV‐1 among domestic pigeons in selected Egyptian governorates. However, the relatively small number of examined birds and the absence of sequence‐based characterization should be considered when interpreting these results. The close interaction between pigeons and backyard poultry may increase the likelihood of cross‐species transmission, emphasizing the importance of continued surveillance, stronger biosecurity measures, and further molecular studies on circulating strains.

## 5. Conclusion

The detection of PPMV‐1 in domestic pigeons from six Egyptian governorates provides preliminary field and laboratory evidence of continued virus circulation in pigeon populations in Egypt. The proximity of pigeons to backyard and commercial poultry, together with their frequent movement through live bird markets, may contribute to virus dissemination and represent an epidemiological concern. Therefore, improved surveillance, strengthened biosecurity, and further molecular characterization of circulating strains are recommended to support future prevention and control programs.

## Author Contributions

Muhammadtaher Abdulrazaq Abdulrasol participated in the planning, laboratory work, molecular detection, data collection, and manuscript drafting. Wafaa A. Abd El‐Ghany contributed to supervision, study design, and critical revision of the manuscript. Harith Abdulla Najem contributed to bacteriological examination, data interpretation, and manuscript revision.

## Funding

The authors did not receive any specific funding for this research. No financial support was provided that could have influenced the study outcomes.

## Disclosure

All authors have read and approved the final version of the manuscript.

## Conflicts of Interest

The authors declare no conflicts of interest.

## Data Availability

The datasets generated and/or analyzed during the current study are available from the corresponding author upon reasonable request.
